# Experimental data on flexural strength of reinforced concrete elements with waste glass particles as partial replacement for fine aggregate

**DOI:** 10.1016/j.dib.2018.03.104

**Published:** 2018-03-28

**Authors:** Olumoyewa Dotun Atoyebi, Obanishola M. Sadiq

**Affiliations:** a Department of Civil Engineering, Landmark University, Omuaran, Kwara State, Nigeria; b Department of Civil & Environmental Engineering, University of Lagos, Lagos, Nigeria

## Abstract

The data in this article are related to the research article titled “Flexural Strength determination of Reinforced Concrete Elements with Waste Glass as Partial Replacement for Fine Aggregate” [1]. The article provides information on reinforced concrete beam elements with the fine aggregate partially replaced with waste glass in proportions of 0%, 10%, 20% and 30%. The beam elements were cured and subjected to flexural load after 7, 14, 28 and 90 days. Three samples were tested for each conditions and the average value computed. The tests records include deflection at each gradual increase in the flexural load and the load at final failure.

**Specifications table**

**Table:** 

Subject area	*Civil Engineering*
More specific subject area	*Construction Materials, Waste Management*
Type of data	*Table, figure*
How data was acquired	*Casting concrete samples in the laboratory and applying flexural load.*
Data format	*Raw*
Experimental factors	*During the curing process, the beam elements were stored in water to reduce the shrinkage effect. The specimens were tested at laboratory conditions.*
Experimental features	*Fine aggregate replaced with waste glass particles to cast beam elements and subjected to flexural load.*
Data source location	*University of Lagos Concrete Laboratory, Yaba, Lagos State. Nigeria.*
Data accessibility	*Data are as presented in this article*
Related Research Article	*Atoyebi Olumoyewa D. (2014) Flexural Strength Determination of Reinforced Concrete Elements with Waste Glass as Partial Replacement for Fine Aggregate. Unpublished MSc Thesis. University of Lagos, Akoka Yaba, Lagos State. Nigeria.*

**Value of the data**

• The data presented shows the response of reinforced concrete elements to flexural load with waste glass as replacement for fine aggregate.
• The data allows for the assessment of the possibility of replacing fine aggregate with waste glass particles.
• The reported data gives information on the effect of waste glass in the reinforced concrete elements on the workability, flexural strength, alkali-silica reaction etc.

## 1 Data

The data presented information on flexural strengths of reinforced concrete element:

• *Deflection value at points of flexural load application on the beam.*
• *Failure load of reinforced concrete beams with fine aggregate replacement at 7, 14, 28 and 90 days of curing.*

## 2 Experimental design, materials and methods

**Fig. 1:**
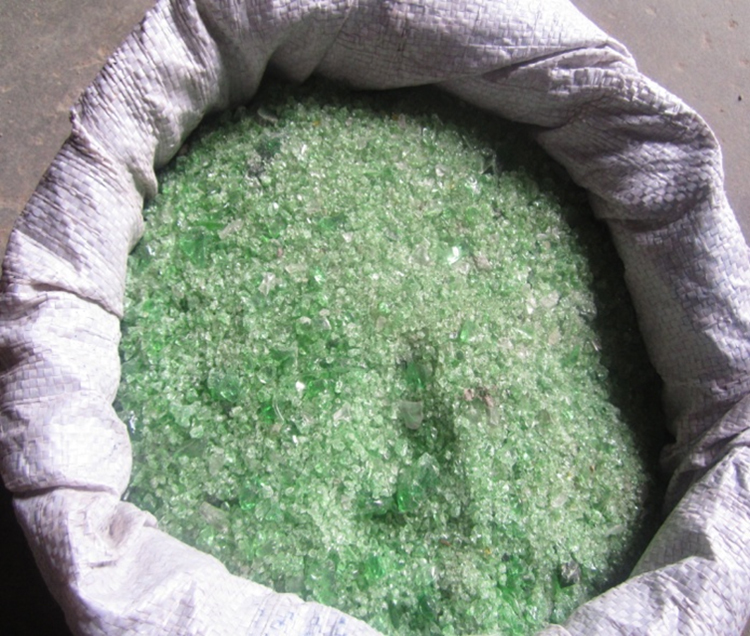
Waste glass particles sample.

**Fig. 2:**
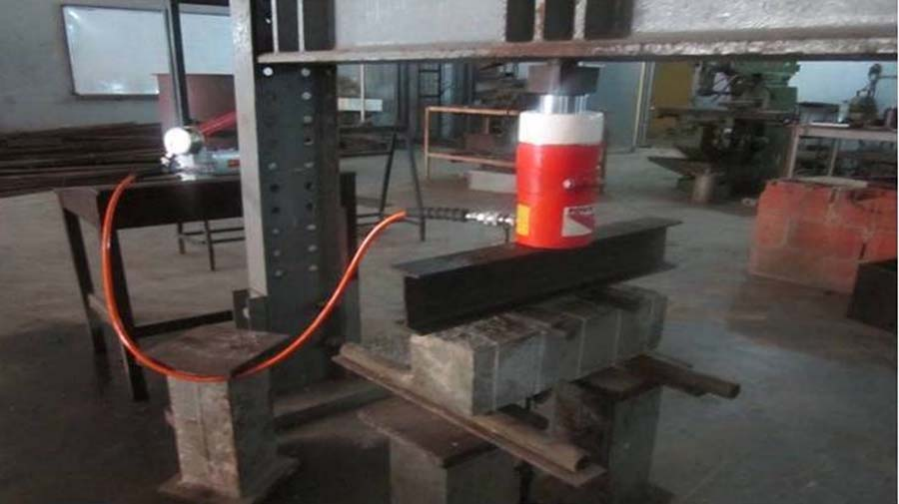
Set up of flexural strength test on beam.

The aggregate materials and cement used for this research were collected from different locations in Lagos state, Nigeria (6.6080°N, 3.6218°E). Waste glass particles (Fig. 1.) collected from Agbara, Ogun State, Nigeria (7° 15′ 0″ North, 3° 24′ 0″ East) was used to partially replace sand as fine aggregate in proportions of 0%,10%,20%and30% [1]. The beams produced were subjected to flexural strength test as shown in Fig. 2 at different curing age. Flexural load and deflection values for 0%, 10%, 20% and 30% replacement at 7 days are presented in Table 1, Table 2, Table 3, Table 4 respectively. Table 5, Table 6, Table 7, Table 8 shows the flexural load and deflection values for 0%,10%,20%and30% replacement at 14 days respectively, Table 9, Table 10, Table 11, Table 12 shows values for 28 days and Table 13, Table 14, Table 15, Table 16 shows values for 90 days for 0%,10%,20%and30% respectively.

**Table 1:** Flexural load and deflection of 0% replacement for 7 days curing.

Load ($\mathrm{kg}/{\mathrm{cm}}^{2})$	Deflection			Average deflection
	Specimen 1	Specimen 2	Specimen 3	
10	6	4	6	5.333333333
20	11	13	12	12
30	17	18	18	17.66666667
40	18	23	21	20.66666667
50	23	26	25	24.66666667
60	27	29	28	28
70	35	31	33	33
80	40	34	38	37.33333333
90	43	38	41	40.66666667
100	47	41	45	44.33333333
110	49	44	46	46.33333333
120	52	47	50	49.66666667
130	54	50	52	52
140	59	54	57	56.66666667
150	63	60	61	61.33333333
160	67	64	66	65.66666667
170	72	69	73	71.33333333
180	76	74	75	75
190	78	76	77	77
200	81	81	82	81.33333333
210	84	85	84	84.33333333
220	86	91	89	88.66666667
230	88	98	95	93.66666667
240	91	103	98	97.33333333
250	95	108	104	102.3333333
260	99			99
270				
280	Failure			
290			Failure	
300		Failure		
Failure	280	300	290	290

Average failure load = 290 $\mathrm{kg}/{\mathrm{cm}}^{2}$

**Table 2:** Flexural load and deflection of 10% replacement for 7 days curing.

Load ($\mathrm{kg}/{\mathrm{cm}}^{2})$	Deflection			Average deflection
	Specimen 1	Specimen 2	Specimen 3	
10	3	5	4	4
20	8	10	11	9.666666667
30	11	14	13	12.66666667
40	16	18	17	17
50	20	21	22	21
60	24	24	25	24.33333333
70	27	25	28	26.66666667
80	30	28	31	29.66666667
90	35	30	33	32.66666667
100	37	32	35	34.66666667
110	40	34	39	37.66666667
120	43	37	42	40.66666667
130	47	39	45	43.66666667
140	50	42	50	47.33333333
150	53	45	52	50
160	57	47	55	53
170	62	50	57	56.33333333
180	65	54	60	59.66666667
190	71	57	63	63.66666667
200	78	64	66	69.33333333
210	83	70	70	74.33333333
220	92	79	81	84
230	104	88	90	94
240		94	96	95
250		105	109	107
260		125		125
270				
280	Failure	Failure		
290			Failure	
300				
Failure	280	280	290	283.3333333

Average failure load = 283.333 $\mathrm{kg}/{\mathrm{cm}}^{2}$

**Table 3:** Flexural load and deflection of 20% replacement for 7 days curing.

Load ($\mathrm{kg}/{\mathrm{cm}}^{2})$	Deflection			Average deflection
	Specimen 1	Specimen 2	Specimen 3	
10	20	7	8	11.66666667
20	25	12	13	16.66666667
30	31	19	18	22.66666667
40	37	23	24	28
50	40	28	29	32.33333333
60	45	31	30	35.33333333
70	48	35	34	39
80	51	38	37	42
90	55	41	42	46
100	58	45	46	49.66666667
110	61	48	50	53
120	65	52	54	57
130	68	56	58	60.66666667
140	72	59	61	64
150	76	63	64	67.66666667
160	80	68	67	71.66666667
170	84	71	72	75.66666667
180	89	75	76	80
190	93	78	79	83.33333333
200	98	83	84	88.33333333
210	100	85	87	90.66666667
220	105	93	94	97.33333333
230		100	107	103.5
240		105	114	109.5
250	Failure	112	117	114.5
260				
270			Failure	
280		Failure		
Failure	250	280	270	266.6666667

Average failure load = 266.67 $\mathrm{kg}/{\mathrm{cm}}^{2}$

**Table 4:** Flexural load and deflection of 30% replacement for 7 days curing.

Load ($\mathrm{kg}/{\mathrm{cm}}^{2})$	Deflection			Average deflection
	Specimen 1	Specimen 2	Specimen 3	
10	8	0	3	3.666666667
20	11	5	7	7.666666667
30	16	8	11	11.66666667
40	19	15	16	16.66666667
50	22	24	19	21.66666667
60	25	28	23	25.33333333
70	30	30	28	29.33333333
80	34	35	32	33.66666667
90	37	37	36	36.66666667
100	40	40	41	40.33333333
110	44	43	44	43.66666667
120	48	47	49	48
130	48	47	49	48
140	48	49	50	49
150	53	56	57	55.33333333
160	58	59	61	59.33333333
170	62	62	63	62.33333333
180	66	65	68	66.33333333
190	70	70	71	70.33333333
200	76	78	75	76.33333333
210	82	84	81	82.33333333
220	92	88	86	88.66666667
230		94	91	92.5
240	Failure		93	93
250				
260				
270		Failure	Failure	
Failure	240	270	270	260

Average failure load = 260 $\mathrm{kg}/{\mathrm{cm}}^{2}$

**Table 5:** Flexural load and deflection of 0% replacement for 14 days curing.

Load ($\mathrm{kg}/{\mathrm{cm}}^{2})$	Deflection			Average deflection
	Specimen 1	Specimen 2	Specimen 3	
10	12	0	4	5.333333333
20	20	4	7	10.33333333
30	24	7	9	13.33333333
40	31	10	13	18
50	36	14	17	22.33333333
60	41	18	20	26.33333333
70	45	22	23	30
80	47	26	27	33.33333333
90	50	29	31	36.66666667
100	55	29	31	38.33333333
110	55	32	34	40.33333333
120	60	38	41	46.33333333
130	63	41	44	49.33333333
140	66	44	47	52.33333333
150	69	46	51	55.33333333
160	73	51	54	59.33333333
170	77	55	57	63
180	82	58	59	66.33333333
190	88	61	62	70.33333333
200	92	65	66	74.33333333
210	97	70	69	78.66666667
220	100	74	73	82.33333333
230	105	78	76	86.33333333
240	110	83	80	91
250	116	86	81	94.33333333
260	117	90	84	97
270		94	90	92
280	Failure	97	93	95
290		103	97	100
300		108	104	106
310		111		111
320			Failure	
330		Failure		
Failure	280	324	320	308

Average failure load = 308 $\mathrm{kg}/{\mathrm{cm}}^{2}$

**Table 6:** Flexural load and deflection of 10% replacement for 14 days curing.

Load ($\mathrm{kg}/{\mathrm{cm}}^{2})$	Deflection			Average deflection
	Specimen 1	Specimen 2	Specimen 3	
10	4	0	2	2
20	9	6	5	6.666666667
30	12	13	9	11.33333333
40	18	19	14	17
50	20	24	19	21
60	23	28	25	25.33333333
70	24	33	29	28.66666667
80	27	38	34	33
90	28	42	36	35.33333333
100	30	45	41	38.66666667
110	31	49	43	41
120	32	52	47	43.66666667
130	34	56	51	47
140	36	59	55	50
150	38	63	57	52.66666667
160	38	68	61	55.66666667
170	40	71	65	58.66666667
180	43	75	68	62
190	45	79	71	65
200	48	84	73	68.33333333
210	52	88	76	72
220	55	93	81	76.33333333
230	58	98	84	80
240	62	102	87	83.66666667
250	66	105	91	87.33333333
260	68	110	95	91
270	74	115		94.5
280	78	118		98
290	83	119	Failure	101
300				
310		Failure		
320	Failure			
Failure	316	310	290	305.3333333

Average failure load = 305.333 $\mathrm{kg}/{\mathrm{cm}}^{2}$

**Table 7:** Flexural load and deflection of 20% replacement for 14 days curing.

Load ($\mathrm{kg}/{\mathrm{cm}}^{2})$	Deflection			Average deflection
	Specimen 1	Specimen 2	Specimen 3	
10	5	1	3	3
20	11	4	7	7.333333333
30	15	8	13	12
40	19	10	16	15
50	22	13	19	18
60	26	14	23	21
70	28	17	26	23.66666667
80	31	20	29	26.66666667
90	34	22	30	28.66666667
100	37	25	34	32
110	39	27	36	34
120	41	31	37	36.33333333
130	44	34	40	39.33333333
140	48	37	42	42.33333333
150	50		45	47.5
160	53		49	51
170	57		53	55
180	61		56	58.5
190	64		60	62
200	69		67	68
210	72		71	71.5
220	74		75	74.5
230	78		80	79
240	84		82	83
250	92		87	89.5
260	96		91	93.5
270	104		96	100
280		FAILURE	99	99
				
290				
300	Failure			
310			Failure	
Failure	300	280	310	296.6666667

Average failure load = 296.667 $\mathrm{kg}/{\mathrm{cm}}^{2}$

**Table 8:** Flexural load and deflection of 30% replacement for 14 days curing.

Load ($\mathrm{kg}/{\mathrm{cm}}^{2})$	Deflection			Average deflection
	Specimen 1	Specimen 2	Specimen 3	
10	6	4	3	4.333333333
20	12	11	9	10.66666667
30	17	16	14	15.66666667
40	21	21	18	20
50	24	25	23	24
60	27	29	27	27.66666667
70	30	33	31	31.33333333
80	33	37	36	35.33333333
90	36	41	44	40.33333333
100	38	44	46	42.66666667
110	41	48	52	47
120	44	50	56	50
130	47	53	59	53
140	50	57	62	56.33333333
150	55	61	64	60
160	58	65	67	63.33333333
170	62	69	68	66.33333333
180	67	73	74	71.33333333
190	74	76	76	75.33333333
200	76	81	80	79
210	81	87	83	83.66666667
220	92	93		92.5
230	95	100		97.5
240	98	111		104.5
250	107	116	Failure	111.5
260		119		119
270		122		122
280				
290	Failure			
300				
310		Failure		
Failure	290	310	250	283.3333333

Average failure load = 283.333 $\mathrm{kg}/{\mathrm{cm}}^{2}$

**Table 9:** Flexural load and deflection of 0% replacement for 28 days curing.

Load ($\mathrm{kg}/{\mathrm{cm}}^{2})$	Deflection			Average deflection
	Specimen 1	Specimen 2	Specimen 3	
10	8	24	6	12.66666667
20	11	33	13	19
30	15	35	17	22.33333333
40	17	41	21	26.33333333
50	21	45	24	30
60	26	49	30	35
70	31	52	34	39
80	34	56	37	42.33333333
90	36	57	41	44.66666667
100	33	61	43	45.66666667
110	36	63	47	48.66666667
120	40	66	54	53.33333333
130	42	69	60	57
140	45	72	64	60.33333333
150	48	75	68	63.66666667
160	52	78	71	67
170	53	80	74	69
180	57	83	76	72
190	59	87	78	74.66666667
200	63	90	81	78
210	66	92	84	80.66666667
220	69	96	87	84
230	74	98	90	87.33333333
240	77	100	96	91
250	81	104	101	95.33333333
260	83	106	103	97.33333333
270	87	109	105	100.3333333
280	93	113	110	105.3333333
290	98	116	117	110.3333333
300	103	121		112
310				
320				
330			Failure	
340		Failure		
350	Failure			
Failure	350	340	330	340

Average failure load = 340 $\mathrm{kg}/{\mathrm{cm}}^{2}$

**Table 10:** Flexural load and deflection of 10% replacement for 28 days curing.

Load ($\mathrm{kg}/{\mathrm{cm}}^{2})$	Deflection			Average deflection
	Specimen 1	Specimen 2	Specimen 3	
10	12	16	11	13
20	23	24	14	20.33333333
30	30	29	18	25.66666667
40	42	35	22	33
50	45	38	24	35.66666667
60	52	43	30	41.66666667
70	58	46	36	46.66666667
80	61	49	39	49.66666667
90	68	52	43	54.33333333
100	73	55	47	58.33333333
110	74	58	54	62
120	76	60	59	65
130	80	62	66	69.33333333
140	84	65	74	74.33333333
150	88	68	79	78.33333333
160	91	70	84	81.66666667
170	96	73	87	85.33333333
180	100	76	91	89
190	104	79	94	92.33333333
200	108	82	98	96
210	113	86	101	100
220	117	90	109	105.3333333
230	122	95	114	110.3333333
240	126	98	116	113.3333333
250	130	104	119	117.6666667
260	140	108	121	123
270		111	126	118.5
280		117	129	123
290		122	131	126.5
300	Failure	115		115
310				
320				
330			Failure	
340		Failure		
Failure	300	340	330	323.3333333

Average failure load = 323.333 $\mathrm{kg}/{\mathrm{cm}}^{2}$

**Table 11:** Flexural load and deflection of 20% replacement for 28 days curing.

Load ($\mathrm{kg}/{\mathrm{cm}}^{2})$	Deflection			Average deflection
	Specimen 1	Specimen 2	Specimen 3	
10	8	4	3	5
20	15	9	7	10.33333333
30	22	12	11	15
40	28	16	14	19.33333333
50	34	18	19	23.66666667
60	38	21	23	27.33333333
70	41	24	29	31.33333333
80	44	25	34	34.33333333
90	48	28	38	38
100	51	30	43	41.33333333
110	53	32	47	44
120	55	34	49	46
130	59	36	54	49.66666667
140	62	38	57	52.33333333
150	64	40	63	55.66666667
160	67	43	68	59.33333333
170	69	46	74	63
180	73	49	78	66.66666667
190	76	51	81	69.33333333
200	78	55	84	72.33333333
210	82	57	87	75.33333333
220	86	60	91	79
230	89	63	93	81.66666667
240	93	66	96	85
250	97	68	99	88
260	100	72	101	91
270	105	76	107	96
280	109	78	108	98.33333333
290		81	111	96
300	Failure	84	114	99
310		87		87
320		90		90
330			Failure	
340				
350		Failure		
Failure	300	350	330	326.6666667

Average failure load = 326.667 $\mathrm{kg}/{\mathrm{cm}}^{2}$

**Table 12:** Flexural load and deflection of 30% replacement for 28 days curing.

Load ($\mathrm{kg}/{\mathrm{cm}}^{2})$	Deflection			Average deflection
	Specimen 1	Specimen 2	Specimen 3	
10	9	5	4	6
20	13	9	8	10
30	20	13	9	14
40	25	16	13	18
50	32	20	19	23.66666667
60	34	22	24	26.66666667
70	35	25	27	29
80	37	27	33	32.33333333
90	40	30	39	36.33333333
100	43	32	46	40.33333333
110	46	34	48	42.66666667
120	48	37	51	45.33333333
130	52	40	54	48.66666667
140	55	41	57	51
150	57	44	61	54
160	60	46	64	56.66666667
170	62	48	66	58.66666667
180	64	51	69	61.33333333
190	69	54	72	65
200	73	56	76	68.33333333
210	76	58	78	70.66666667
220	79	59	81	73
230	82	62	83	75.66666667
240	85	64	86	78.33333333
250	88	67	89	81.33333333
260	92	69	91	84
270	93	71	94	86
280	98	76	97	90.33333333
290	104	78	100	94
300	108	81	105	98
310	120	92	113	108.3333333
320		124	116	120
330				
340	Failure			
350			Failure	
360		Failure		
Failure	345	360	350	351.6666667

Average failure load = 351.667 $\mathrm{kg}/{\mathrm{cm}}^{2}$

**Table 13:** Flexural load and deflection of 0% replacement for 90 days curing.

Load ($\mathrm{kg}/{\mathrm{cm}}^{2})$	Deflection			Average deflection
	Specimen 1	Specimen 2	Specimen 3	
25	25	13	19	19
50	37	32	28	32.33333333
75	45	41	36	40.66666667
100	65	45	49	53
125	76	56	61	64.33333333
150	84	64	77	75
175	94	68	95	85.66666667
200	98	74	105	92.33333333
225	105	81	109	98.33333333
250	110	83	117	103.3333333
275	121	91	126	112.6666667
300	125	95	131	117
325	142	103	139	128
350	148	109	145	134
375	156	115	147	139.3333333
400	163	123	154	146.6666667
425		129	162	145.5
450	Failure	136		136
475			Failure	
500		Failure		
Failure	452	500	476	476

Average failure load = 476 $\mathrm{kg}/{\mathrm{cm}}^{2}$

**Table 14:** Flexural load and deflection of 10% replacement for 90 days curing.

Load ($\mathrm{kg}/{\mathrm{cm}}^{2})$	Deflection			Average deflection
	Specimen 1	Specimen 2	Specimen 3	
25	8	12	13	11
50	29	27	24	26.66666667
75	38	39	36	37.66666667
100	43	52	49	48
125	53	55	56	54.66666667
150	60	58	61	59.66666667
175	62	62	66	63.33333333
200	68	66	70	68
225	72	73	74	73
250	78	77	77	77.33333333
275	89	81	85	85
300	94	86	90	90
325	98	94	96	96
350	105	100	110	105
375	113	105	114	110.6666667
400	118	117	119	118
425	126	121	126	124.3333333
450	134	127	131	130.6666667
475	Failure	136	138	137
500			Failure	
525				
550		Failure		
Failure	452	547	500	499.6666667

Average failure load = 499.667 $\mathrm{kg}/{\mathrm{cm}}^{2}$

**Table 15:** Flexural load and deflection of 20% replacement for 90 days curing.

Load ($\mathrm{kg}/{\mathrm{cm}}^{2})$	Deflection			Average deflection
	Specimen 1	Specimen 2	Specimen 3	
25	10	11	13	11.33333333
50	24	18	19	20.33333333
75	34	22	25	27
100	40	27	31	32.66666667
125	45	32	38	38.33333333
150	49	34	43	42
175	54	38	50	47.33333333
200	57	41	59	52.33333333
225	61	47	65	57.66666667
250	67	53	69	63
275	73	59	71	67.66666667
300	77	63	75	71.66666667
325	82	72	79	77.66666667
350	88	79	81	82.66666667
375	93	88	85	88.66666667
400	102	94	96	97.33333333
425	110	103	107	106.6666667
450		112	110	111
475	Failure	Failure		
500			Failure	
Failure	452	452	476	460

Average failure load = 460 $\mathrm{kg}/{\mathrm{cm}}^{2}$

**Table 16:** Flexural load and deflection of 30% replacement for 90 days curing.

Load ($\mathrm{kg}/{\mathrm{cm}}^{2})$	Deflection			Average deflection
	Specimen 1	Specimen 2	Specimen 3	
25	11	19	11	13.66666667
50	19	22	18	19.66666667
75	25	29	24	26
100	27	32	30	29.66666667
125	33	37	32	34
150	37	43	34	38
175	42	45	43	43.33333333
200	46	49	47	47.33333333
225	50	60	51	53.66666667
250	56	60	53	56.33333333
275	61	64	62	62.33333333
300	68	67	68	67.66666667
325	73	75	76	74.66666667
350	78	82	79	79.66666667
375	84	87	88	86.33333333
400	90	92	94	92
425	96	99	103	99.33333333
450	Failure	106	112	109
475		Failure		
500			Failure	
Failure	428	452	476	452

Average failure load = 452 $\mathrm{kg}/{\mathrm{cm}}^{2}$				
