# Supplementary material for: Experimental data on flexural strength of reinforced concrete elements with waste glass particles as partial replacement for fine aggregate
Source: Data Brief. 2018 Mar 28;18:846–59. doi: 10.1016/j.dib.2018.03.104 (PMC5996735; doi:10.1016/j.dib.2018.03.104)
Supplement: Supplementary file 1 — Supplementary material [file mmc1.docx]

Declaration of Interest

This research work is part of an MSc Thesis of Atoyebi Olumoyewa Dotun and there is no specific grant from funding agencies in the public, commercial, or non profiting sectors.
